# *Limosilactobacillus fermentum* WIS32 alleviates LPS-induced depression-like behavior via the microbiota-gut-brain axis

**DOI:** 10.3389/fmicb.2026.1792026

**Published:** 2026-05-19

**Authors:** Xiaohan Li, Ping Li, Yanan Yang, Feng Cai, Ying Cao, Jiaguo Zhan, Jinqiang Zhu, Yunfeng Duan, Chongming Wu

**Affiliations:** 1 School of Chinese Materia Medica, Tianjin University of Traditional Chinese Medicine, Tianjin, China; 2 PKUMed-Wisbiom Joint Laboratory for Human Microbiome Research, Beijing, China; 3 State Key Laboratory of Chinese Medicine Modernization, Tianjin, China; 4 Tianjin Key Laboratory of Therapeutic Substance of Traditional Chinese Medicine, Tianjin, China

**Keywords:** depression, microbiota-gut-brain axis, gut microbiota, probiotics, *Limosilactobacillus fermentum*

## Abstract

**Introduction:**

In recent years, the global incidence of depression has continued to rise, posing a severe threat to physical and mental health as well as overall quality of life. While *Lactobacillus*, as a significant probiotic, has demonstrated remarkable potential in alleviating depressive symptoms, the specific mechanisms of *Limosilactobacillus fermentum* in depression have not been fully elucidated. This study aimed to evaluate the antidepressant effects of *L. fermentum* WIS32 and explore its underlying mechanisms via the microbiota-gut-brain axis (MGBA).

**Methods:**

A cross-cohort meta-analysis was initially conducted to assess the abundance of *L. fermentum* in the gut microbiota of patients with depression. Subsequently, in vitro experiments were performed to evaluate the gastrointestinal tolerance, antioxidant capacity, and antibacterial properties of WIS32 compared to a control strain, LFG89. In vivo, a lipopolysaccharide (LPS)-induced mouse model of depression was established to assess the effects of WIS32 intervention on behavioral phenotypes, hippocampal neurochemicals (5-HT and BDNF), and serum pro-inflammatory cytokines (TNF-α, IL-1β, and IL-6). Finally, full-length 16S rRNA sequencing and correlation analyses were utilized to examine the remodeling of the gut microbiota structure.

**Results:**

The meta-analysis revealed a significant reduction in the abundance of *L. fermentum* in patients with depression. In vitro assessments demonstrated that WIS32 exhibited superior gastrointestinal tolerance, antioxidant capacity, and antibacterial properties compared to LFG89. In vivo, WIS32 intervention significantly ameliorated LPS-induced depressive-like behaviors, upregulated 5-HT and BDNF levels in the hippocampus, and inhibited serum pro-inflammatory cytokines. Furthermore, 16S rRNA sequencing indicated that WIS32 significantly remodeled the gut microbiota by promoting beneficial taxa, such as *Bacteroides stercorirosoris* and *Massilimaliae timonensis*, while inhibiting pro-inflammatory pathogens like *Streptococcus*—effects that were more pronounced than those of LFG89. Correlation analysis confirmed that these microbial shifts were strongly associated with reduced inflammation and enhanced neuroplasticity.

**Discussion:**

These findings demonstrate that *L. fermentum* WIS32 effectively alleviates depressive symptoms by modulating key taxa within the gut microbiota to suppress inflammation and promote neuroplasticity. Therefore, *L. fermentum* WIS32 holds significant promise as a potential psychobiotic for the treatment and management of depression.

## 1 Introduction

Depression is a common and severe mental disorder that carries a significant global disease burden. According to data from the World Health Organization, the global prevalence of depression is approximately 4.4%, affecting over 300 million people, and the incidence has continued to rise in recent years (Zhou et al., 2025). Clinically, patients with depression typically present with persistent low mood, loss of interest, insomnia or hypersomnia, changes in appetite, and difficulty concentrating; severe cases may even involve suicidal ideation or behavior (Malhi and Mann, 2018). Current research into the pathogenesis of depression involves multifaceted factors, primarily including imbalances in monoamine neurotransmitters, decreased expression of brain-derived neurotrophic factor (BDNF), impaired neuroplasticity in the limbic system, and circadian rhythm dysregulation (Hasler, 2010). Due to the complex and diverse etiologies of depression, its treatment remains a challenge. Although selective serotonin reuptake inhibitors (SSRIs) are widely used, only about 28% of patients achieve remission within 8 weeks, and adverse effects often lead to treatment discontinuation (Trivedi et al., 2006). While psychotherapy is effective for mild-to-moderate cases, it is time-consuming and its efficacy varies (Park and Zarate, 2019; Cuijpers et al., 2014). Given the limitations of existing treatment regimens, it is of critical importance to explore safe alternative therapies that can target the multidimensional pathological features of depression.

With the deepening of microbiome research, the mechanistic links between gut microbiota and depression have become increasingly clear, offering novel perspectives for overcoming traditional therapeutic challenges (Su et al., 2024; Wang et al., 2024; Duan et al., 2022). A growing body of evidence highlights the critical role of the microbiota-gut-brain axis in depression. The gut microbiota engages in bidirectional communication with the central nervous system through neural, endocrine, and immune pathways (Ojeda et al., 2021). Disruption of this interaction can compromise intestinal barrier integrity, promote systemic inflammatory responses, and lead to neuropsychiatric disorders, including depression (Eltokhi and Sommer, 2022). Patients with depression exhibit significant alterations in gut microbiota, with distinct differences in microbial composition compared to healthy controls. Specifically, the abundance of beneficial bacteria, such as *Faecalibacterium* and *Bifidobacterium*, is significantly reduced, while pro-inflammatory taxa, such as *Prevotella* and the family *Enterobacteriaceae*, are markedly increased. These changes correlate significantly with the severity of depressive symptoms (Jiang et al., 2015). To further explore these mechanisms, researchers have established various experimental models, among which the lipopolysaccharide (LPS)-induced depression model is widely utilized. Subsequent studies have found that intraperitoneal injection of LPS triggers a systemic inflammatory response, leading to microglial activation and the subsequent release of neurotoxins. This process reduces synaptic plasticity and impairs intestinal barrier function, establishing a persistent cycle of inflammatory response and neurological dysfunction that ultimately results in cognitive impairment and behavioral abnormalities (Qin et al., 2007; Zhao et al., 2019; Zhang et al., 2022; Mamunur et al., 2023; Zhu et al., 2017).

Given the close association between the gut microbiota and depression, along with its underlying pathological mechanisms, microecological intervention strategies centered on probiotics have demonstrated significant potential. Research indicates that probiotic supplementation can alleviate depressive symptoms by modulating microbial balance, with Lactobacillus becoming a focal point of study due to its high correlation with the microbiota characteristics of affected patients (He et al., 2025). Existing evidence suggests that *Limosilactobacillus* may exert antidepressant effects by regulating the gut microbiota and enhancing immune responses (Choi et al., 2025). For instance, *Agropyron smithii* enriched with *Limosilactobacillus* effectively alleviates lipopolysaccharide (LPS)-induced depressive behavior (Chang et al., 2024). *Lactobacillus delbrueckii* has been shown to reduce hippocampal inflammation and increase BDNF levels by inhibiting the TLR4/NF-κB pathway (Qiu et al., 2021). Furthermore, *Lactobacillus helveticus* NS8 supplementation significantly improves depressive-like behavior, modulates the function of the hypothalamic–pituitary–adrenal (HPA) axis, and increases the abundance of beneficial microbiota (Alatan et al., 2024). *Lactobacillus casei Shirota* (LcS) not only regulates stress hormones, neurotransmitters, and blood–brain barrier function in animal models, but clinical trials also demonstrate its ability to significantly relieve anxiety and somatic symptoms (Takada et al., 2016). These findings not only confirm the dynamic and persistent interaction between the gut microbiota and the central nervous system (CNS) but also elucidate the core regulatory mechanisms of the “microbiota-gut-brain axis” in depression at the molecular level.

Although significant progress has been made in the study of the genus *Lactobacillus*, there remains a conspicuous gap in research regarding the mechanisms of its important subpopulation, *Limosilactobacillus fermentum*. As a Gram-positive, non-spore-forming *lactic acid bacterium* (LAB), this species is widely distributed in the human gut and various fermented foods. In models of depression, *L. fermentum* ATCC 9338 can regulate HPA axis function and synaptic plasticity, thereby alleviating depressive-like behaviors (Tiwari and Paramanik, 2025). Furthermore, *L. fermentum* HNU312 effectively mitigates abnormal brain development and behavioral disorders induced by early-life lead exposure by reducing oxidative damage and improving synaptic plasticity (Zhang et al., 2023). Despite these explorations, existing findings are primarily based on phenotypic observations, and systematic analyses of neurotransmitter regulation and immune-neural interactions remain insufficient. Significant heterogeneity exists among different strains regarding their mechanisms and efficacy, yet precise strain-screening methods targeting depression are lacking. The antidepressant activity and specific molecular mechanisms of the WIS32 strain remain unclear, limiting its clinical application potential.

The primary objective of this study was to investigate the antidepressant effects of WIS32 and to elucidate its mechanisms of action through the microbiota-gut-brain axis (MGBA). First, a meta-analysis was conducted to compare the alterations of *Limosilactobacillus fermentum* in the gut of patients with depression versus healthy individuals. Subsequently, a novel strain, *L. fermentum* WIS32, was isolated from the breast milk of healthy volunteers. *In vitro* experiments were then performed to compare the gastrointestinal tolerance, antioxidant capacity, and antibacterial properties of WIS32 with those of the control strain LFG89, thereby evaluating the functional differences between these probiotics. In a lipopolysaccharide (LPS)-induced mouse model of depression, we assessed behavioral indicators, neurochemical markers, and inflammatory response outcomes. Furthermore, full-length 16S rRNA sequencing technology was utilized to analyze the impact of WIS32 on gut microbiota composition and to explore the correlations between microbial community shifts and depression-related biomarkers. This study aims to provide a theoretical foundation for the application of WIS32 in the treatment of depression through microbial ecological regulation.

## 2 Materials and methods

### 2.1 Individual-based meta-analysis of gut microbiota in depressed patients

To systematically obtain gut microbiota data from patients with depression, we conducted a comprehensive search of the PubMed and Web of Science databases using the keywords “Depression,” “Major depressive disorder,” and “gut microbiota.” Based on this, we screened for original research articles published in English that had publicly available raw sequencing data of gut microbiota in public databases. Subsequently, we batch retrieved and downloaded the corresponding FASTQ files in a Linux operating system environment. Perform quality control, noise reduction, and species annotation on each obtained dataset, and ultimately generate an operational taxonomic unit (OTU) table for further in-depth analysis (Zou et al., 2024).

### 2.2 Culture and preparation of *Limosilactobacillus fermentum*

#### 2.2.1 Bacterial strain and culture conditions

The *Limosilactobacillus fermentum* WIS32 used in this study was isolated from a breast milk sample provided by a healthy volunteer at the mature milk stage (approximately 30 days postpartum). The donor was a 28-year-old healthy female with no history of metabolic, psychiatric, or infectious diseases, and had not consumed antibiotics or probiotic supplements within the 3 months prior to sampling. The sample collection was performed under strict aseptic conditions with the donor’s written informed consent. After screening and identification, the target strains were preserved in a glycerol stock. For experimental use, strain WIS32 and the commercial reference strain LFG89 were, respectively, streaked onto sterile MRS agar plates and incubated under anaerobic conditions at 37 °C for 48 h. Subsequently, single colonies were picked and transferred into MRS broth for further cultivation. Following two successive rounds of subculturing (secondary activation) to ensure optimal viability and transition into the logarithmic growth phase, the strains were utilized for subsequent experiments.

#### 2.2.2 Preparation of bacterial suspension for oral gavage

The test strain WIS32 and the control strain LFG89 (provided by Beijing Wisbiom Biotechnology Co., Ltd. and MGBlab) were inoculated into MRS liquid medium at a ratio of 2% (v/v) and cultured at 37 °C for 24 h to obtain the fermentation broth. The bacterial cells were collected by centrifugation (6,000 × g, 5 min), and after discarding the supernatant, the pellets were resuspended in sterile saline. The final concentration of the bacterial suspension was adjusted to 5 × 10^9^ CFU/mL for subsequent animal gavage experiments.

### 2.3 Evaluation of probiotic properties in *Limosilactobacillus fermentum* strains

#### 2.3.1 *In vitro* gastrointestinal survival assay

The simulated gastrointestinal digestion model was established following the methodology of a previous study (Minekus et al., 2014). Artificial gastric juice (containing pepsin, pH 2.5 ± 0.1) and artificial intestinal juice (containing pancreatin, pH 6.8 ± 0.1) were prepared, sterilized using a 0.22 μm membrane filter, and stored at 4 °C. A 10 mL volume of the bacterial suspension was centrifuged (6,000 × g, 10 min), washed twice, and resuspended in the aforementioned digestive juices to a final volume of 10 mL. After thorough mixing, the samples were incubated anaerobically at 37 °C for 3 h. The number of viable bacteria before treatment (N_0_) and after treatment (N_1_) was determined using the MRS plate count method. The survival rate was calculated according to the following formula: Survival rate (%) = [log_10_(N_1_) /log_10_(N_0_)] × 100.

#### 2.3.2 Analysis of antioxidant properties of bacterial isolates

WIS32 and LFG89 were inoculated at 5% (v/v) into 50 mL of MRS medium and subjected to three consecutive generations of activation culture at 36 °C (24 h per generation). After centrifugation of the culture broth, the cell-free supernatant was obtained. The bacterial pellets were washed twice with pre-cooled PBS and adjusted to a concentration of 1 × 10^9^ CFU/mL prior to ultrasonic disruption. The resulting lysate was centrifuged and filtered through a 0.22 μm microporous membrane to prepare the bacterial extract. Finally, the DPPH radical scavenging capacity (Nanjing Jiancheng Bioengineering Institute, A153-1-1), hydroxyl radical scavenging capacity (Nanjing Jiancheng Bioengineering Institute, YX-W-A505), and total antioxidant capacity (Nanjing Jiancheng Bioengineering Institute, A015-3-1) of each sample were detected in strict accordance with the manufacturer’s instructions.

#### 2.3.3 *In vitro* evaluation of antibacterial performance

The antibacterial activity of the strains was determined using the agar well diffusion method. Six common pathogenic bacteria were selected and inoculated into BHI liquid medium at a ratio of 1% (v/v) and cultured at 37 °C for 16 h. Once the absorbance of the bacterial suspension was adjusted to OD_600_ = 0.025, it was mixed with BHI agar medium pre-warmed to 50 ± 1 °C to prepare double-layer plates. After the upper layer of agar solidified, Oxford cups were placed aseptically on the surface, and 200 μL of WIS32 or LFG89 fermentation supernatant (processed via centrifugation and filtration) was added to each cup; an equal volume of sterilized MRS liquid medium served as the blank control. The plates were first refrigerated at 4 °C for 24 h to allow for diffusion, followed by incubation at 37 °C for 24 h. Finally, the diameter of the inhibition zones was measured and recorded using a vernier caliper to evaluate the antibacterial efficacy of each strain.

### 2.4 Animal experiments

Forty 8-week-old male SPF Kunming mice (purchased from Beijing HFK Bioscience Co., Ltd.) were used in this study. All mice were housed in an air-conditioned room within the animal center (22 °C–24 °C, 40–50% relative humidity, 12:12 h light/dark cycle) with ad libitum access to food and water. All procedures were conducted in accordance with institutional guidelines and approved by the Ethics Committee of Tianjin University of Traditional Chinese Medicine (Approval No.: TCM-LAEC2023083).

To evaluate the antidepressant effects of *WIS32*, a mouse model of depression was induced using lipopolysaccharide (LPS, Sigma-Aldrich, USA), with the LPS dosage determined based on the study by Chang et al. (2024). Mice were randomly assigned to five groups: the control group (Norm), the model group (LPS), the positive drug group (Positive, fluoxetine 3 mg/kg) (Yang and Wei, 2025), the test strain group (WIS32), and the control strain group (LFG89). During the 14-day pretreatment period prior to induction, the Norm and LPS groups were administered an equal volume of saline (0.2 mL/mouse) daily by oral gavage, The Positive group received daily intragastric administration of fluoxetine solution (3 mg/kg) as a positive control, and the WIS32 and LFG89 groups were gavaged daily with their respective bacterial suspensions (5 × 10^9^ CFU/mL, 0.2 mL/mouse).

**Figure 1:**
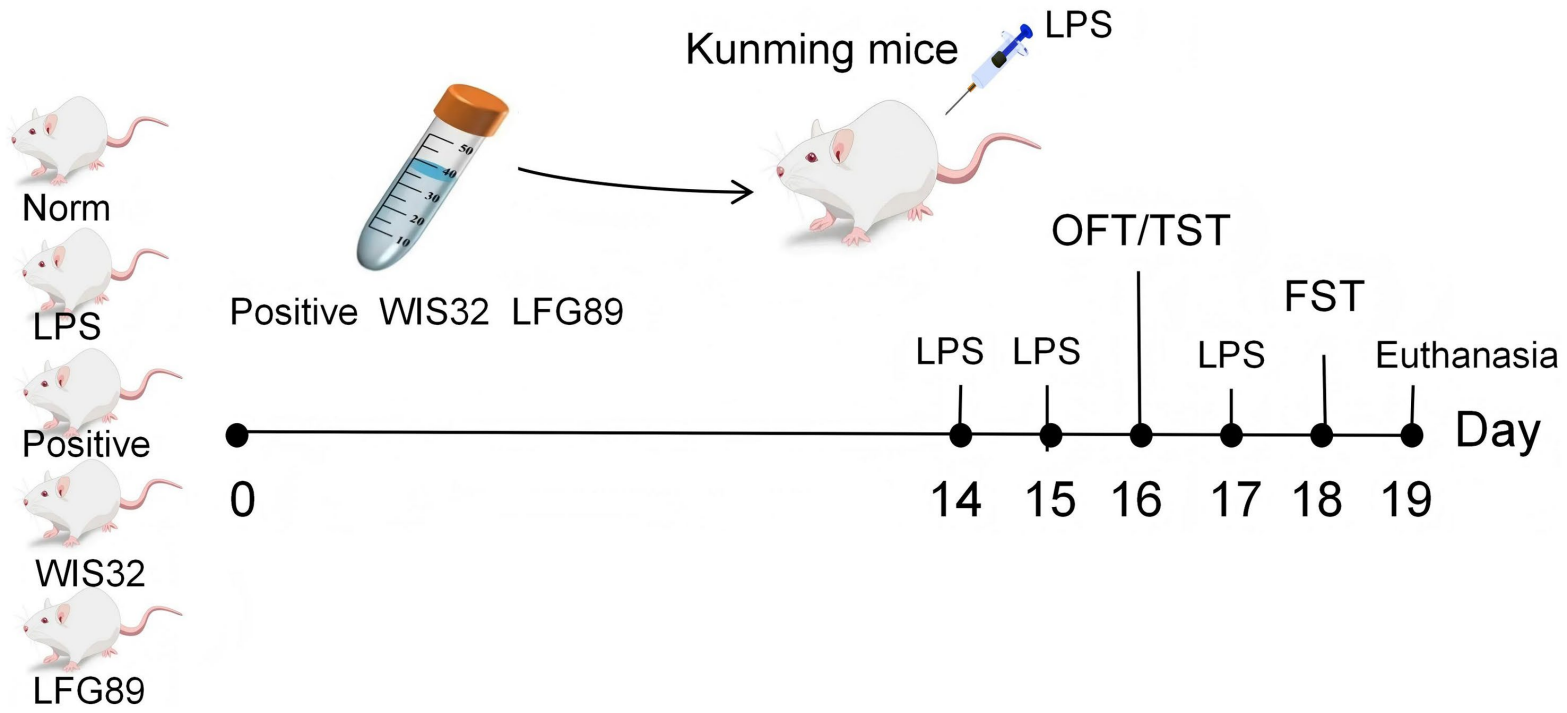
Experimental timeline of the experimental design and behavioral test protocol.

On days 14 and 15 of the experiment, all groups except the Norm group received intraperitoneal injections of LPS (1 mg/kg) for two consecutive days to establish the depression model. On day 16, the open field test (OFT) and tail suspension test (TST) were conducted. On day 17, an additional intraperitoneal injection of LPS was administered, followed by the forced swimming test (FST) 24 h later on day 18. To ensure the objectivity of the experimental results, all behavioral tests and data recording were performed using a single-blind method. The experimental personnel responsible for observing, recording, and calculating the ‘immobility time’ of the mice were blinded to the grouping information, and the statistical analysis was independently completed by another researcher who was also unaware of the experimental grouping. During the period of LPS injections and behavioral testing, each treatment group continued to maintain the original administration intervention. To monitor changes in the intestinal microbiota, fresh feces were collected on the morning of day 19 following the conclusion of the intervention. Mice were placed in clean, dry cages lined with sterile filter paper to collect naturally discharged fresh feces (no less than 100 mg per mouse). To prevent contamination, sterile forceps were used during the collection process, and samples were immediately stored in sterile cryovials, rapidly frozen in liquid nitrogen, and finally stored in an ultra-low temperature freezer at −80 °C for subsequent analysis. Subsequently, the mice were euthanized, and serum and hippocampal tissues were collected for follow-up detection (Figure 1).

### 2.5 Behavioral evaluation

#### 2.5.1 Open field test

The Open Field Test (OFT) was conducted on the morning of experimental day 16. Prior to the experiment, mice were transferred to the behavioral laboratory 3 h in advance to acclimate to the environment and were allowed to adapt within the open field test box for 1 h before formal testing. During the test, each mouse was placed in the center of a square open field box (100 cm × 100 cm × 40 cm), and its subsequent movement trajectory and stay time in the central area were recorded and filmed for 5 min (Savignac et al., 2016). The experiment was performed in a dark and quiet environment. To eliminate the influence of light and odors on subsequent tests, the box was thoroughly cleaned with 75% ethanol after each mouse was tested. The central area was defined as the middle 1/4 of the floor area, and the time spent in this region was used to assess the anxiety levels of the mice.

#### 2.5.2 Tail suspension test

The Tail Suspension Test (TST) was performed on the afternoon of day 16 (Xu et al., 2023). Mice were transported to the laboratory environment 1 h prior to the experiment. During the test, each mouse’s tail was secured using medical tape (12 cm long and 3 cm wide), maintaining a distance of 2–3 mm from the tip of the tail. The handling time for each mouse was strictly controlled within 10 s. The other end of the tape was fixed to a suspension bracket, ensuring the snout of the inverted mouse was approximately 30 cm above the ground. The duration of the experiment was 6 min, and the cumulative immobility time during the final 4 min was recorded.

#### 2.5.3 Forced swimming test

The Forced Swim Test (FST) was conducted on day 18 (24 h after the final LPS injection). Each mouse was placed in a cylindrical glass tank (30 cm height, 18 cm diameter, 20 cm water depth) with the water temperature maintained at 22–25 °C (Can et al., 2012). The water depth ensured that the mouse could not touch the bottom while its head remained above the surface. Each mouse was allowed to swim for a total of 6 min, with the first 2 min serving as an acclimation period and the cumulative immobility time during the final 4 min being manually recorded.

### 2.6 Tissue preparation

After anesthetizing the mice with a single dose of 2% sodium pentobarbital (40 mg/kg; Sigma-Aldrich, USA), blood samples were collected from the orbital venous plexus, followed by cervical dislocation. The blood was allowed to stand for 2 h and then centrifuged at 4,000 rpm for 10 min at 4 °C to separate and extract the serum. Immediately after blood collection, the mice were perfused through the left ventricle with ice-cold 0.01 M PBS buffer (pH 7.4), after which the whole brain was rapidly removed and the bilateral hippocampus (HPC) was isolated on ice. The hippocampal tissue was added to ice-cold PBS buffer (containing 1% protease and phosphatase inhibitors; Keygen Biotech, Nanjing) at a weight-to-volume ratio of 1:9 (w/v) and thoroughly homogenized. The homogenate was centrifuged at 10,000 rpm for 10 min at 4 °C, and the supernatant was collected. The total protein concentration in the supernatant was determined using a BCA protein assay kit (Solarbio, Beijing). Samples were then aliquoted and stored in a −80 °C freezer for subsequent analysis.

### 2.7 ELISA-based measurement of 5-HT, BDNF, and inflammatory markers

According to the manufacturer’s instructions, enzyme-linked immunosorbent assay (ELISA) kits were employed to determine the levels of TNF-*α* (Solarbio, SEKM-0034), IL-1β (Solarbio, SEKM-0002), IL-6 (Solarbio, SEKM-0007), 5-HT (Solarbio, EEL006), and BDNF (Solarbio, EEL088) in the previously prepared serum and hippocampal tissue homogenates. The detection results for hippocampal tissues were normalized to total protein concentration. The absorbance of each well was measured at a wavelength of 450 nm using a microplate reader.

### 2.8 16S rRNA sequencing

Fecal DNA was extracted according to established protocols. The full-length 16S rRNA gene (V1–V9 regions) was amplified using the primers 27F (5′-AGAGTGTGATCCTGGCTCAG−3′) and 1492R (5′-TACGGCTACCTTGTACGACTT−3′). Amplicons were extracted from 2% agarose gels and purified to construct SMRTbell libraries (Pacific Biosciences). Sequencing was performed by Shanghai Biozeron Biotechnology Co., Ltd. (Shanghai, China) on the PacBio Sequel II platform. Raw FASTA reads underwent high-quality filtering and alignment. Operational Taxonomic Units (OTUs) were clustered at a 97% similarity threshold. Subsequent analyses, including relative abundance, alpha-diversity, and beta-diversity, were conducted using R software (version 4.2.3).

Phylogenetic analysis of *L. fermentum* WIS32 was conducted by aligning its full-length 16S rRNA sequence with 12 NCBI reference strains via ClustalW. The phylogenetic tree was generated in MEGA 7.0 using the Neighbor-Joining (NJ) algorithm and the Maximum Composite Likelihood (MCL) model. Branch robustness was assessed with 1,000 bootstrap iterations.

### 2.9 Statistical analysis

Statistical analysis was performed using GraphPad Prism 10.1.2 software. Experimental data are expressed as Mean ± SEM. Comparisons between three or more groups were conducted using one-way analysis of variance (one-way ANOVA). Statistical assessments were further evaluated using the Brown-Forsythe test and Welch’s test (ANOVA) to determine the significance of differences between groups. A value of *p* < 0.05 was considered statistically significant.

## 3 Results

### 3.1 Patients with depression exhibit a significant reduction in *Limosilactobacillus fermentum*

To elucidate the intrinsic association between gut microbiota composition and the pathogenesis of depression, we analyzed the gut microbiota characteristics of depression patients and healthy individuals based on the available 16S rRNA gene sequencing datasets. Through literature screening on the PubMed database, we ultimately included 4 relevant studies, and the raw sequencing data provided by these 4 studies can be accessed via NCBI BioProject, with the BioProject numbers being PRJNA776170, PRJNA830325, PRJNA687871, and PRJNA838414.

**Figure 2:**
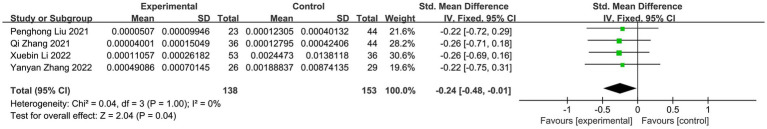
Forest plot illustrating the meta-analysis of patients with depression.

Our comparative analysis of microbial abundance between depression patients and healthy controls (Figure 2) revealed a significant reduction in the abundance of *Limosilactobacillus fermentum* in depression patients. This cross-cohort meta-analysis revealed that the reduction of *Limosilactobacillus fermentum* is a common feature among depression patients, suggesting that *Limosilactobacillus fermentum* plays a critical role in maintaining gut health in depression patients.

### 3.2 Identification and *in vitro* probiotic characterization of strain WIS32

**Figure 3:**
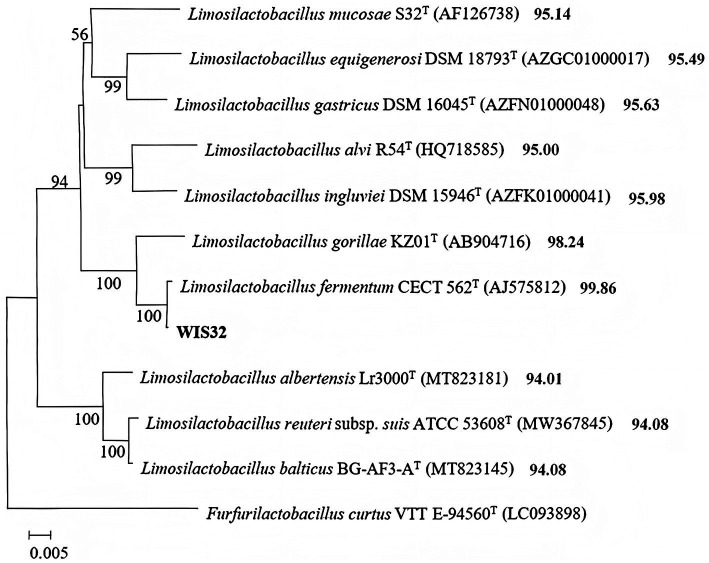
Phylogenetic tree based on 16S rRNA gene sequences, showing the evolutionary relationship of WIS32 with other *Limosilactobacillus* species. The tree was constructed using the neighbor-joining method with 1,000 bootstrap replicates.

Phylogenetic analysis was performed based on the full-length 16S rRNA gene sequences of closely related *Limosilactobacillus* species (Figure 3). The Neighbor-Joining (NJ) tree revealed that WIS32 clustered robustly within the *Limosilactobacillus fermentum* lineage, forming a well-supported clade with the type strain *L. fermentum* CECT 562ᵀ, supported by a bootstrap value of 100%. Evolutionary distances calculated using the Maximum Composite Likelihood (MCL) model indicated minimal divergence within this clade (sequence identity of 99.86%), confirming that WIS32 falls within the species boundaries of *L. fermentum*. Furthermore, phylogenetic analysis clearly demonstrated a distinct genetic distance between WIS32 and related species such as *L. gorillae*, *L. reuteri*, and *L. albertensis*, thereby precisely defining its taxonomic status.

To evaluate the tolerance of the two *Limosilactobacillus fermentum* strains, LFG89 and WIS32, in simulated gastric and intestinal fluids, the dominant strain was screened by measuring bacterial survival rates (Tables 1, 2). After treatment with artificial gastric juice at pH 2.5 for 3 h, the survival rates of both strain WIS32 and the control strain LFG89 were below 10%. However, statistical analysis revealed that the survival rate of WIS32 (7.21% ± 0.65%) was significantly higher than that of LFG89 (3.78% ± 0.42%) (*n* = 3, *p* < 0.05). This indicates that WIS32 possesses a stronger capacity to tolerate gastric acid stress (Table 1). The survival performance of the strains was further evaluated after 3 h of treatment in an artificial intestinal fluid environment. Results showed that both WIS32 and the control strain LFG89 exhibited good tolerance. Specifically, the survival rate of WIS32 (88.02% ± 2.45%) was significantly higher than that of the control strain LFG89 (69.25% ± 3.12%) (*n* = 3, *p* < 0.01) (Table 2). Although both strains showed higher survival rates in the intestinal environment compared to the gastric environment, strain WIS32 demonstrated exceptional stability.

**Table 1:** Survival rates of LFG89 and WIS32 in simulated gastric fluid at pH 2.5. (*n* = 3, *p < 0.05, **p < 0.01, ***p < 0.001).

Strain number	Simulated gastric fluid 0 h (CFU/mL)	Simulated gastric fluid 3 h (CFU/mL)	Survival rate (Mean ±SEM, %)	*p* value (vs. LFG89)
LFG89	1.10 ± 0.12 × 10^9^	4.14 ± 0.35 × 10^7^	3.78 ± 0.42	
WIS32	2.66 ± 0.21 × 10^9^	1.92 ± 0.18 × 10^8^	7.21 ± 0.65*	0.018

**Table 2:** Survival rates of LFG89 and WIS32 in artificial intestinal fluid at pH 6.8. (*n* = 3, *p < 0.05, **p < 0.01, ***p < 0.001).

Strain number	Simulated intestinal fluid 0 h (CFU/mL)	Simulated intestinal fluid 3 h (CFU/mL)	survival rate (Mean ±SEM, %)	*p* value (vs. LFG89)
LFG89	3.74 ± 0.27 × 10^9^	2.59 ± 0.19 × 10^9^	69.25 ± 3.12	
WIS32	1.64 ± 0.14 × 10^9^	1.46 ± 0.11 × 10^9^	88.02 ± 2.45**	0.0042

Regarding the evaluation of probiotic functions, both WIS32 and the control strain LFG89 exhibited certain *in vitro* antioxidant activities, as determined by measuring DPPH radical scavenging capacity, hydroxyl radical scavenging capacity, and total antioxidant capacity (Table 3). In terms of DPPH radical scavenging capacity, the clearance rate of the WIS32 fermentation supernatant reached 77.57% ± 1.65%, which was significantly superior to that of the control strain LFG89 (72.09% ± 1.42%, *p* < 0.05). For hydroxyl radical (-OH) scavenging capacity, the WIS32 fermentation supernatant demonstrated a highly significant advantage, with a clearance rate as high as 37.26% ± 2.15%, far exceeding that of LFG89 (7.23% ± 0.95%, *p* < 0.01). Although LFG89 showed a relatively higher performance in the assessment of total antioxidant capacity (T-AOC), the comprehensive results indicate that the metabolites of WIS32 are superior to the control strain in scavenging specific free radicals.

**Table 3:** Antioxidant capacities of *Limosilactobacillus fermentum* and their metabolites (*n* = 3, *p < 0.05, **p < 0.01, ***p < 0.001, ****p < 0.0001).

Strain name	DPPH radical scavenging ability		-OH radical scavenging ability		total antioxidant capacity (equivalent of mM FeSO₄)	
	Bacterial extract	Fermentation broth supernatant	Bacterial extract	Fermentation broth supernatant	Bacterial extract	Fermentation broth supernatant
*LFG89*	39.24 ± 1.52	72.09 ± 1.42	41.57 ± 2.31	7.23 ± 0.95	0.1253 ± 0.0082	0.5750 ± 0.021
*WIS32*	40.52 ± 1.85	77.57 ± 1.65*	37.64 ± 2.05	37.26 ± 2.15**	0.0860 ± 0.0054	0.4880 ± 0.0185

Furthermore, in the evaluation of antibacterial effects (Table 4), both WIS32 and LFG89 exhibited inhibitory capabilities against *Cronobacter sakazakii* and *Pseudomonas aeruginosa*. Statistical analysis revealed that the inhibition zone diameter of WIS32 was significantly larger than that of LFG89 (*p* < 0.05), confirming its superior antibacterial activity. Compared to LFG89, WIS32 demonstrated stronger inhibitory effects against the aforementioned pathogens, further validating its enhanced antibacterial potential.

**Table 4:** Antibacterial efficacy of WIS32 fermentation supernatant against pathogenic bacteria.

Pathogenic bacteria	Inhibition zone (mm)	
	WIS32	LFG89
*Cronobacter sakazakii*	11.72 ± 0.29*	10.24 ± 0.47
*Staphylococcus aureus*	—	—
*Pseudomonas aeruginosa*	10.41 ± 0.81*	8.69 ± 0.41
*Shigella flexneri*	—	—
*Enterococcus faecalis*	—	—
*Escherichia coli*	—	—
Control	—	—

The following values represent the diameter of the inhibition zone, in mm. “—” indicates no antimicrobial activity. The diameter of the Oxford cup is 7.8 mm.*indicates that, compared with the LFG89 group, the difference in the inhibition zone diameter of WIS32 against the pathogenic bacterium is statistically significant (*P* < 0.05).

### 3.3 WIS32 alleviates LPS-induced depressive-like behaviors in mice

**Figure 4:**
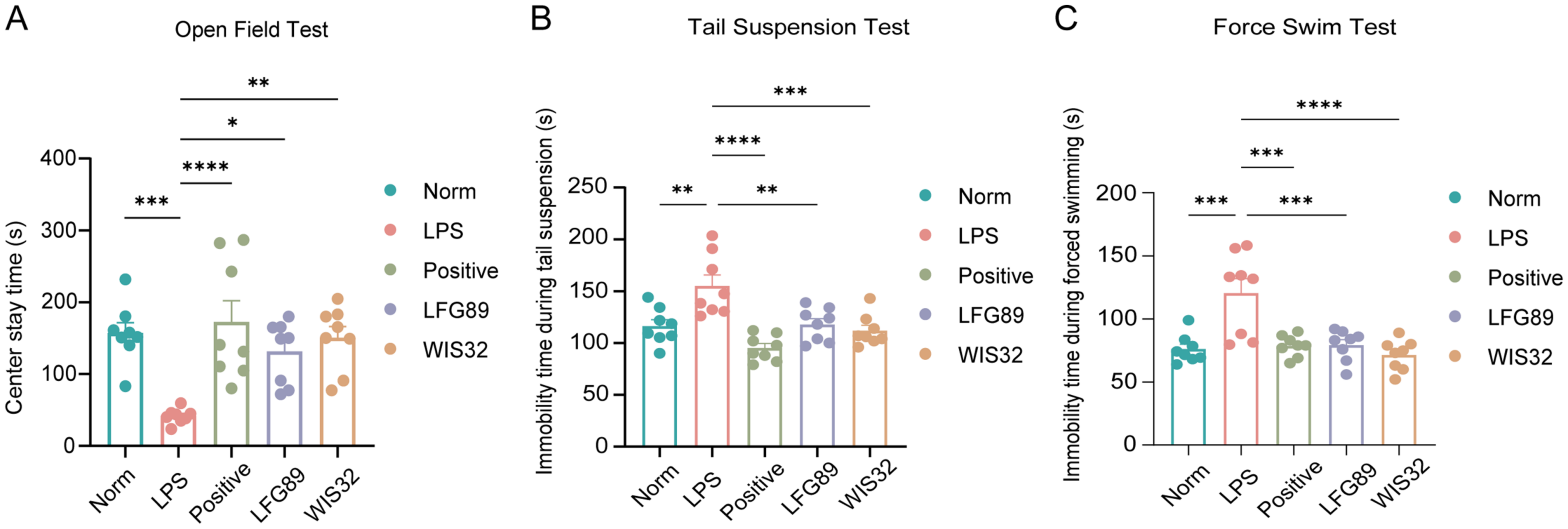
WIS32 attenuates lipopolysaccharide (LPS)-induced depressive-like behaviors in mice. **(A)** Center residence time in mice; immobility time during tail suspension **(B)** and forced swimming **(C)**. *p < 0.05, **p < 0.01, ***p < 0.001, ****p < 0.0001.

To evaluate depression and anxiety-related disorders through behavioral indicators, we observed behavioral changes in depressed mice during the Open Field Test (OFT), Tail Suspension Test (TST), and Forced Swim Test (FST), and assessed the therapeutic intervention of WIS32. The results showed that, compared with the normal group (Norm), the LPS group (LPS) exhibited a significant reduction in the time spent in the central area during the OFT (Figure 4A, *p* < 0.001), and a significant increase in immobility time during both the TST and FST (Figures 4B,C, *p* < 0.001). These findings indicate that LPS successfully induced depressive-like behaviors in the mice. Intervention with WIS32 significantly reversed these LPS-induced behavioral abnormalities, as evidenced by a significant increase in the time spent in the central area during the OFT (Figure 4A, *p* < 0.01) and a substantial decrease in immobility time during the TST (Figure 4B, *p* < 0.001) and FST (Figure 4C, *p* < 0.0001). The efficacy of WIS32 showed a similar trend to that of the positive control group treated with fluoxetine. These findings demonstrate that WIS32 plays a pivotal role in alleviating LPS-induced depression and associated symptoms in mice.

### 3.4 WIS32 upregulates hippocampal neurotransmitters and neurotrophic factors and mitigates neuroinflammation

**Figure 5:**
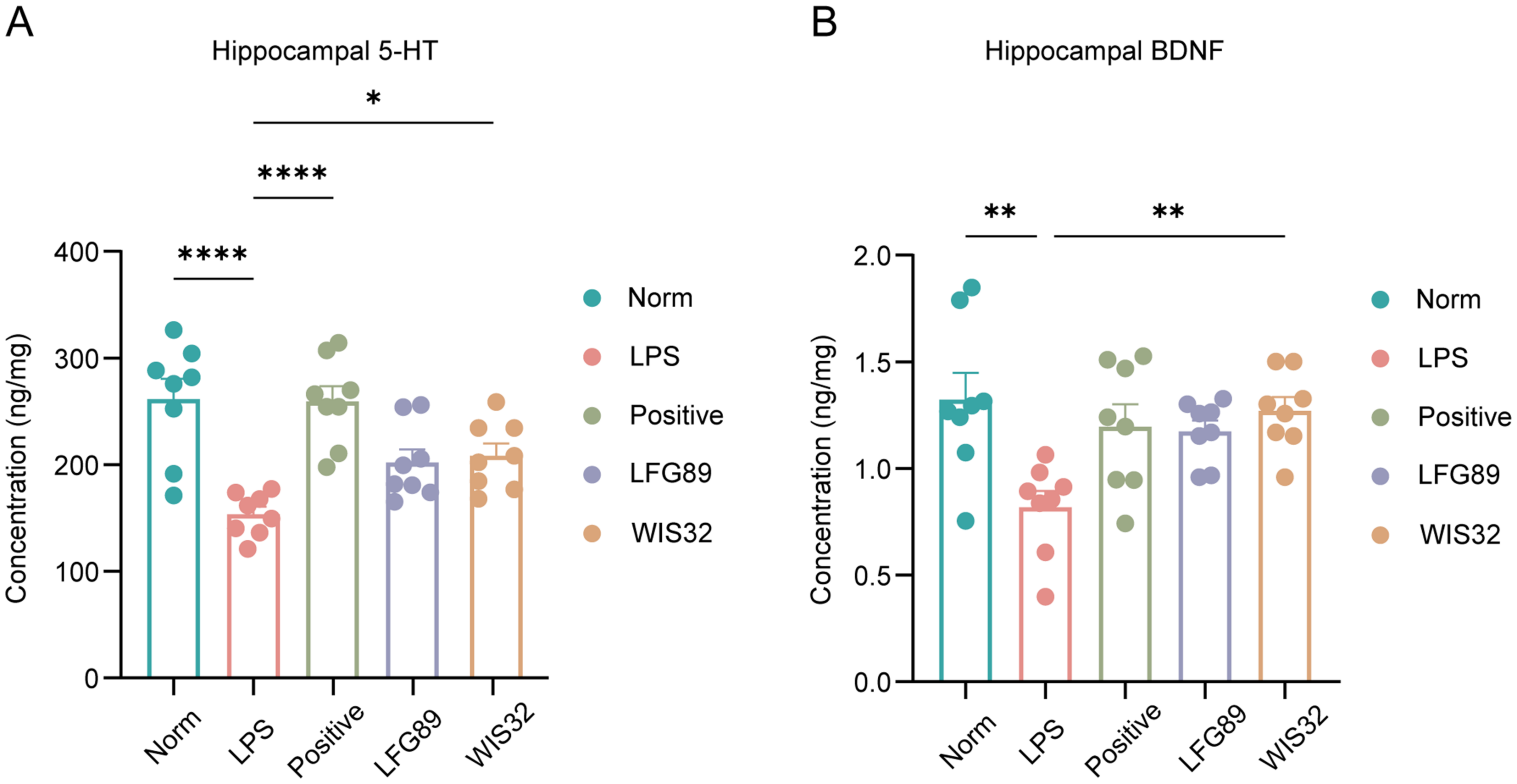
WIS32 restores LPS-induced decline in hippocampal neurotransmitters: **(A)** Hippocampal levels of 5-HT and **(B)** BDNF. ^*^p < 0.05, **p < 0.01, ***p < 0.001, ****p < 0.0001.

To further validate the antidepressant effects of WIS32 in LPS-induced mice, we measured the levels of 5-hydroxytryptamine (5-HT) and brain-derived neurotrophic factor (BDNF) in the hippocampus. The results demonstrated that LPS treatment significantly reduced the levels of hippocampal 5-HT (Figure 5A, *p* <0.0001) and BDNF (Figure 5B, *p* < 0.01). Following WIS32 intervention, 5-HT levels significantly recovered (*p* < 0.05), and BDNF levels were also significantly reversed (*p* < 0.01), with the improvement being superior to that of the LFG89 control group.

**Figure 6:**
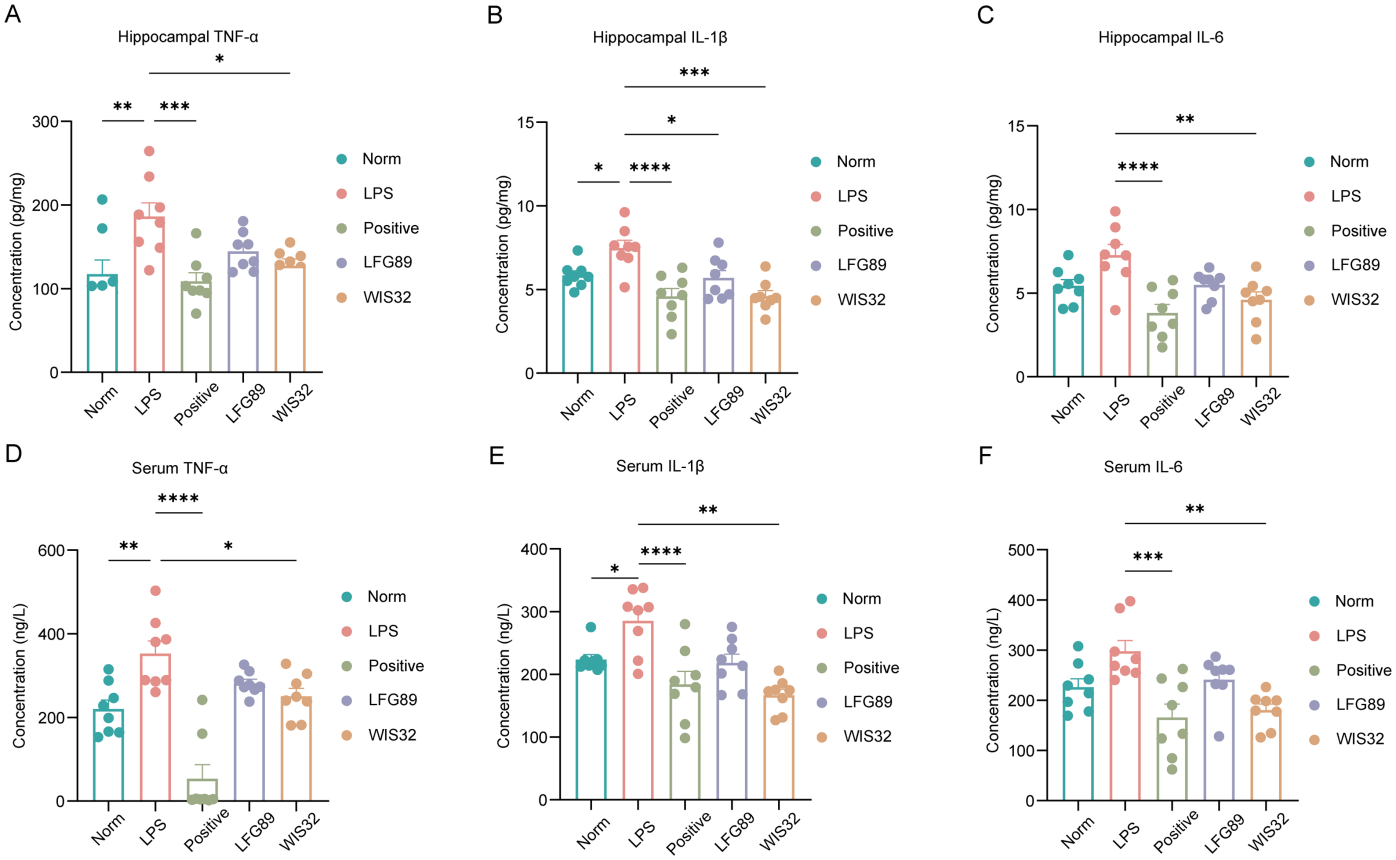
WIS32 prevents LPS-induced increase in hippocampal and serum inflammatory factors. **(A–C)** Represent the levels of hippocampal inflammatory factors TNF-*α*, IL-1β, and IL-6; **(D–F)** represent the levels of serum inflammatory factors TNF-α, IL-1β, and IL-6. *p < 0.05, **p < 0.01, ***p < 0.001, ****p < 0.0001.

Furthermore, we examined changes in peripheral and central inflammatory cytokines. LPS administration significantly increased the levels of TNF-*α*, IL-1β, and IL-6 in both the hippocampus and serum (Figure 6). Notably, WIS32 intervention exhibited significant anti-inflammatory activity. In hippocampal tissues, WIS32 significantly decreased the expression of TNF-α (*p* < 0.05) and IL-6 (*p* < 0.01), and its effect on downregulating IL-6 was significantly better than that of the LFG89 control group (*p* < 0.01). In peripheral serum, IL-1β levels in the WIS32 group were significantly lower than those in the model group (*p* < 0.01), showing an effect consistent with the positive control (Positive) group treated with fluoxetine.

### 3.5 WIS32 reshapes the gut microbial community in LPS-induced mice

**Figure 7:**
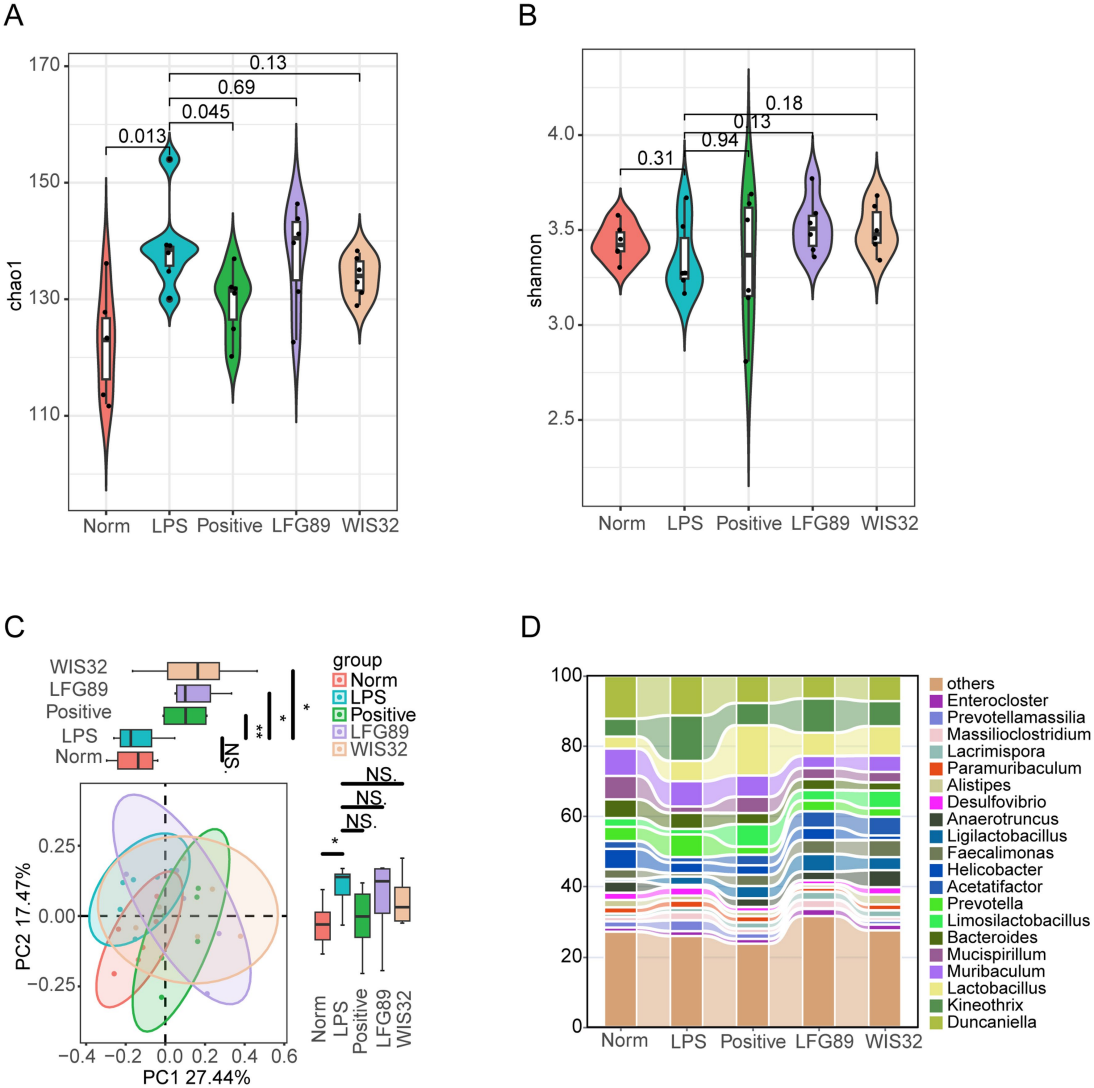
The effect of WIS32 on gut microbial diversity and structure. The α-diversity of gut microbiota was assessed by **(A)** Chao1 and **(B)** Shannon indices. **(C)** The structure of gut microbiota is represented by the PCoA plot. **(D)** Relative abundance of gut microbiota at the genus levels. NS not significant. *p < 0.05, **p < 0.01.

Gut microbiota dysbiosis influences the development of depression through bidirectional communication via the microbiota-gut-brain axis (MGBA). Therefore, we analyzed the effects of *Limosilactobacillus fermentum* WIS32 on the gut microbiota of LPS-induced depressed mice. The Chao1 index, used to evaluate the alpha-diversity of the gut microbiota, showed a significant increase in the LPS group compared to the normal (Norm) group (Figure 7A, *p* < 0.05). Compared with the LPS group, the positive intervention group restored the alpha-diversity of the gut microbiota, while no significant changes were observed in the other two groups. There were no significant differences in the Shannon index among the five groups (Figure 7B). Principal Coordinate Analysis (PCoA) based on beta-diversity revealed a significant separation in microbial structure between the LPS group and the Norm group (*p* < 0.01), indicating that LPS administration disrupted the gut microbiota structure. The WIS32 intervention group also showed significant separation from the LPS group in PCoA space (Figure 7C, *p* < 0.05). At the genus level, compared with the Norm group, the LPS group exhibited a decrease in the abundance of *Mucispirillum*, *Limosilactobacillus*, *Acetatifactor*, *Faecalimonas*, *Anaerotruncus*, and *Alistipes*, alongside an increase in the abundance of *Kineothrix*, *Prevotella*, *Desulfovibrio*, *Paramuribaculum*, *Massilioclostridium*, and *Prevotellamassilia*. WIS32 intervention was able to reverse these trends. Notably, both the LPS and WIS32 groups showed a decrease in the abundance of *Helicobacter*, *Duncaniella*, *Muribaculum*, and *Bacteroides*, and an increase in *Ligilactobacillus*, *Lacrimispora*, *Enterocloster*, and *Lactobacillus*; however, these changes were more pronounced in the WIS32 group (Figure 7D). These results demonstrate that WIS32 effectively remodels the structure of the gut microbial community in mice.

### 3.6 WIS32 alleviates depressive symptoms via specific modulation of gut microbiota correlated with behavioral improvement

**Figure 8:**
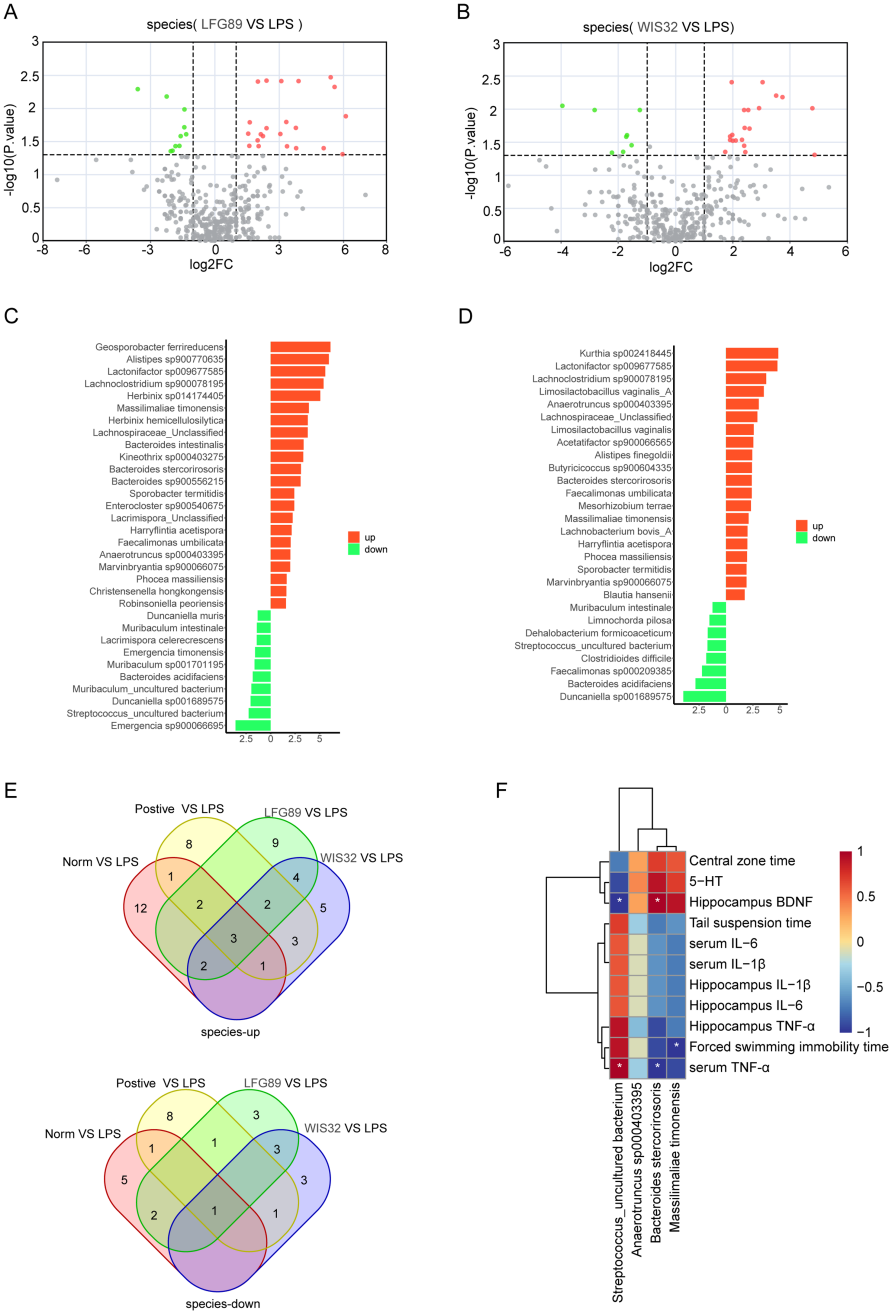
Correlation between differential microbial species and phenotypes. **(A)** Volcano plot analysis of differences in gut microbiota genera between the *LFG89* and model groups. **(B)** Volcano plot analysis of differences in gut microbiota genera between the *WIS32* and model groups. **(C)** Bar chart showing the fold changes in gut microbiota genera between the *LFG89* and model groups. **(D)** Bar chart showing the fold changes in gut microbiota genera between the *WIS32* and model groups. **(E)** Venn diagram showing the upregulated and downregulated species in gut microbiota across group comparisons. **(F)** Correlation between gut microbiota and biochemical indicators. Blue indicates negative correlations, red indicates positive correlations. *p < 0.05, **p < 0.01.

To further identify the key bacteria potentially involved in the antidepressant effects of WIS32, an inter-group analysis of microbial composition was performed. The results showed that compared with the LPS group, 22 bacterial species were upregulated in the LFG89 group, including *Lachnoclostridium* sp900078195, unclassified *Lachnospiraceae*, *Bacteroides stercorirosoris*, and *Massilimaliae timonensis*; meanwhile, 10 species were downregulated, such as *Enterocloster* spp., *Emergencia* sp900066695, and uncultured *Streptococcus* (Figures 8A,C). In the WIS32-treated depressed mice, 20 bacterial species significantly increased and 8 species significantly decreased (Figure 8B). Among these, *Lachnoclostridium* sp900078195, unclassified *Lachnospiraceae*, *Lactobacillus amylovorus* A, *Limosilactobacillus vaginalis* A, *Limosilactobacillus vaginalis*, *Anaerotruncus* sp000403395, *Bacteroides stercorirosoris*, and *Massilimaliae timonensis* were upregulated, while *Intestinimonas butyriciproducens* and uncultured *Streptococcus* were downregulated (Figure 8D).

At the species level, compared with the LPS group, three species were commonly upregulated and one species was commonly downregulated across the Norm, Positive, LFG89, and WIS32 groups (Figure 8E). Correlation analysis between biochemical indicators and gut microbiota revealed that uncultured *Streptococcus* was positively correlated with inflammatory cytokines (TNF-*α*, IL-1β, and IL-6) in both serum and hippocampal tissues, as well as with immobility time in the TST and FST (*r* > 0.6). Specifically, it showed a significant positive correlation with serum TNF-α (*r* > 0.6, *p* < 0.05). Simultaneously, it was negatively correlated with hippocampal 5-HT, BDNF, and central area stay time in the OFT, with a significant negative correlation observed for hippocampal BDNF (*r* < −0.6,*p* < 0.05). Conversely, *Anaerotruncus* sp000403395, *Bacteroides stercorirosoris*, and *Massilimaliae timonensis* were positively correlated with hippocampal 5-HT, BDNF, and OFT central stay time, and negatively correlated with inflammatory cytokines and immobility times. Notably, *Bacteroides stercorirosoris* exhibited a significant negative correlation with serum TNF-α (*r* < −0.6, *p* < 0.05) and a significant positive correlation with hippocampal BDNF (*r* > 0.6, *p* < 0.05). Furthermore, *Massilimaliae timonensis* showed a significant negative correlation with immobility time in the FST (*r* < −0.6, *p* < 0.05) (Figure 8F). In summary, WIS32 exerts its antidepressant effects by remodeling the gut microbiota structure, inhibiting inflammatory responses, and enhancing neural plasticity.

## 4 Discussion

Currently, the clinical treatment of depression relies primarily on antidepressant medications; however, monotherapy is characterized by low cure rates and high recurrence rates. Therefore, it is imperative to explore methods for enhancing the therapeutic efficacy of depression treatments. As safe and widely recognized dietary supplements, probiotics have demonstrated potential value as adjunctive therapies for depression. Increasing clinical evidence suggests that probiotics can alleviate symptoms of depression and anxiety by modulating the “microbiota-gut-brain axis” (MGBA) (Bravo et al., 2011; Wang et al., 2019).

This study confirms that intraperitoneal injection of lipopolysaccharide (LPS) significantly elevates the levels of TNF-*α*, IL-1β, and IL-6 in the mouse hippocampus. From a pathophysiological perspective, LPS acts as an exogenous stressor capable of triggering a systemic inflammatory response by activating the NLRP3/Caspase-1/ASC inflammasome pathway in hippocampal microglia. Activated microglia serve as the primary source of pro-inflammatory cytokines in the brain. The neurotoxins they release further reduce synaptic plasticity and impair neuronal function, establishing a vicious cycle of inflammation and neurological dysfunction that ultimately leads to the manifestation of depressive-like behaviors (Kim et al., 2023).

*Limosilactobacillus fermentum* is a lactic acid bacterium with significant probiotic properties, widely prevalent in the human gastrointestinal tract and traditional fermented foods. Its characteristics include strong acid and bile salt tolerance and high adhesion (Pan et al., 2011), which enable it to successfully bypass the gastrointestinal environment and colonize intestinal epithelial cells to form a protective barrier. This strain exerts health benefits—such as antioxidant, anti-inflammatory, lipid-lowering, and metabolic improvement effects—through mechanisms including the regulation of gut microbiota balance, inhibition of pathogenic bacterial growth, enhancement of intestinal barrier function, and immunomodulation (Naghmouchi et al., 2020). Research on *L. fermentum* in the medical field has primarily focused on metabolic diseases (e.g., obesity) and its adjunctive role in the immunomodulation of IBD (Huang et al., 2025; Liang et al., 2025; Li et al., 2019). However, research regarding its impact on neurological disorders, including depression, remains in the preliminary stages. Our experimental results demonstrated that WIS32 improved the gut microbiota and that its intervention significantly reduced the expression levels of TNF-α and IL-6 in hippocampal tissues. This suggests that WIS32 may indirectly inhibit the overactivation of hippocampal glial cells by modulating the microbiota-gut-brain axis (Li et al., 2025). Compared with the control strain LFG89, WIS32 exhibited a more significant advantage in downregulating IL-6. This precise downregulation of central inflammatory markers provides the basis for the improvement of immobility time observed in mice during the tail suspension test (TST) and forced swim test (FST).

Although both *Lactobacillus* and *Bifidobacterium* are common probiotics, their colonization capacities in the gut differ significantly: *Bifidobacterium longum* exhibits robust colonization, whereas *Lactobacillus casei* is more easily cleared from the intestinal tract (de Albuquerque et al., 2018; Xiao et al., 2021). We screened the dominant strain WIS32 and compared it with the control strain LFG89, finding that WIS32 maintained a higher survival rate than LFG89 after 3 h in artificial gastric juice. Both strains demonstrated excellent tolerance after 3 h in artificial intestinal fluid, with WIS32 performing superiorly. Furthermore, WIS32 exhibited distinct free radical scavenging and antioxidant capacities. This strain also showed inhibitory effects against *Cronobacter sakazakii* and *Pseudomonas aeruginosa*, with higher potency than LFG89. These characteristics are of significant value for the probiotic development of the WIS32 strain. *Lacticaseibacillus rhamnosus* GG, recognized for its high acid and bile tolerance, can stably survive and adhere within the intestine, thereby repairing the intestinal barrier and reducing the spread of systemic inflammation to the central nervous system (Chivero et al., 2022; Segers and Lebeer, 2014). The aforementioned studies confirm that the acid tolerance, bile resistance, and favorable growth characteristics of probiotics facilitate their survival in the gut, enabling them to exert therapeutic effects on the host. Therefore, we further investigated the probiotic effects of *Limosilactobacillus fermentum* and found that the WIS32 strain possesses superior acid resistance, antioxidant capacity, and antibacterial properties. These characteristics facilitate its colonization and functional performance within the intestinal tract. This suggests that metabolites produced by WIS32 in the gut may directly enter the systemic circulation through the intestinal mucosa, or alternatively, block the transmission of peripheral inflammatory signals to the central nervous system by alleviating localized intestinal oxidative stress and inflammation.

The occurrence of depression is often closely associated with dysfunctional neurotransmitter systems, particularly abnormal changes in hippocampal neurotransmitter levels, which are considered key factors in the pathogenesis of the disorder. *Limosilactobacillus fermentum* can significantly promote the secretion of 5-hydroxytryptamine (5-HT) and brain-derived neurotrophic factor (BDNF) in depressed mice. This result is consistent with the findings reported by Wang, which demonstrated that *L. fermentum* NS9 regulates hippocampal 5-HT and BDNF levels by producing indole derivatives, thereby significantly improving antibiotic-induced anxiety and depressive-like behaviors in mice (Wang et al., 2015).

Furthermore, the role of inflammatory responses in the pathogenesis of depression has garnered widespread attention. The aforementioned NS9 strain has also been shown to improve antibiotic-induced anxiety and depressive-like behaviors by inhibiting the activation of hippocampal microglia and the secretion of inflammatory cytokines (such as IL-6 and TNF-*α*) in the serum. Fluoxetine, a commonly used antidepressant, has been proven to reduce serum levels of inflammatory markers IL-1*β*, IL-6, and TNF-α, thereby alleviating depressive symptoms (Mojiri-Forushani et al., 2023). This evidence suggests that inhibiting inflammatory responses contributes to the mitigation of depressive symptoms. Similarly, we found that WIS32 significantly reduced the levels of TNF-α, IL-1β, and IL-6 in both the serum and hippocampus, indicating that the improvement of depression by WIS32 may be related to the inhibition of inflammatory cytokine secretion and the promotion of neurotransmitter release.

In recent years, with the in-depth exploration of the “microbiota-gut-brain axis” theory, probiotics have demonstrated unique interventional potential in the treatment of depression by modulating the gut microbiota. The gut microbiota interacts with the brain through pathways involving the vagus nerve, the immune system, and neurotransmitters (Kaur et al., 2023). For example, in Alzheimer’s disease (AD) model mice, *Faecalibacterium prausnitzii* can significantly reduce amyloid-β (Aβ) deposition and neuroinflammatory responses while improving cognitive function by restoring gut microbiota balance (Ueda et al., 2021). Colonization with *Akkermansia muciniphila* can increase intestinal 5-HT levels, further alleviating depressive symptoms by altering gut-brain signaling (Guo et al., 2024). This evidence indicates that specific probiotics possess potential therapeutic efficacy in neurodegenerative diseases and depression by regulating gut microbiota equilibrium and improving gut-brain signaling.

To this end, we investigated the changes in the gut microbiota of depressed mice following intervention with *Limosilactobacillus fermentum* and explored the correlations between key microbial regulation, hippocampal neurotransmitters, and inflammatory cytokines. Although no significant differences in gut microbiota alpha-diversity were observed after *L. fermentum* intervention compared to the LPS group, the overall microbial structure was altered following treatment with WIS32 and LFG89. In the WIS32 group, the relative abundances of *Bacteroides stercorirosoris*, *Massilimaliae timonensis*, and *Anaerotruncus* sp000403395 were significantly increased, while the relative abundance of uncultured *Streptococcus* was significantly decreased. Our study found that uncultured *Streptococcus* was significantly positively correlated with inflammatory cytokines in both the hippocampus and serum. Certain species of the genus *Streptococcus* have been proven to possess the potential to penetrate the blood–brain barrier and trigger central inflammatory responses (Orihuela et al., 2009). By significantly reducing the abundance of this pathogen, WIS32 inhibits the infiltration of Iba1^+^/CD11b^+^ cells (activated microglia) into the hippocampus and induces hippocampal BDNF expression, thereby alleviating depression. *Anaerotruncus* sp000403395, *B. stercorirosoris*, and *M. timonensis* were significantly positively correlated with 5-HT and BDNF levels, as well as with the time spent in the central area during the OFT. These species were negatively correlated with the levels of TNF-*α*, IL-1β, and IL-6 in the serum and hippocampal tissue, as well as with the immobility time in the TST and FST. *Anaerotruncus colihominis*, which belongs to the same genus as *Anaerotruncus* sp000403395, can significantly alleviate symptoms of experimental autoimmune encephalomyelitis by inducing RORγt ^+^ regulatory T cells in mesenteric lymph nodes; its abundance is negatively correlated with disease severity, indicating its potential to inhibit neuroinflammation (Bianchimano et al., 2022). The genus *Bacteroides* is a major producer of gamma-aminobutyric acid (GABA) in the gut, which can inhibit excessive neuronal excitation through the gut-brain axis, thereby relieving symptoms of anxiety and depression (Conn et al., 2024).

The aforementioned research indicates that the antidepressant mechanism of *Limosilactobacillus fermentum* lies not only in the regulation of peripheral immunity but also in its ability to remodel the gut microbiota structure. By reducing circulating endotoxins produced by pro-inflammatory bacteria, it alleviates hippocampal glia-mediated neuroinflammatory responses through microbiota-gut-brain axis signaling. This multi-dimensional regulation significantly enhances neural plasticity, increases BDNF levels, and restores the balance of monoamine neurotransmitters, specifically 5-HT (Qi et al., 2023).

This study has certain limitations, and future research offers several promising directions. First, we did not validate the antidepressant effects of WIS32 in germ-free mice to further clarify the central contribution of the gut microbiota. A deeper exploration of the molecular mechanisms of WIS32 will facilitate a better understanding of its antidepressant actions and provide a theoretical basis for the treatment of LPS-induced depression. Although this study reveals the remodeling effect of WIS32 on the microbiota structure, it has not yet directly identified the specific molecular biomarkers entering the systemic circulation through metabolomic approaches. Future research will integrate non-targeted metabolomic analysis to further clarify whether the antidepressant effects of WIS32 stem from the absorption of its own specific metabolites or are achieved through the secondary metabolic signals generated by the modulation of the gut microbiota. Research has shown that multi-strain probiotics can exert neuroprotective effects by remodeling the gut microbiota composition and promoting the proliferation of short-chain fatty acid (SCFA)-producing bacteria. These bacteria activate the AKT/GSK-3β signaling pathway, effectively inhibiting Tau protein hyperphosphorylation, regulating tryptophan metabolism balance, promoting 5-HT production, and reducing neurotoxic kynurenine (Kyn) levels while alleviating neuroinflammation through the inhibition of the NF-κB pathway (Xiao-hang et al., 2024). Additionally, *γ*-aminobutyric acid (GABA) released by *Lacticaseibacillus paracasei* can transmit anti-inflammatory signals via the vagus nerve, enhancing the resistance of neuronal cells to oxidative damage (Ferrari et al., 2023). Indole-3-lactic acid (ILA) produced by *Bifidobacterium* reduces IL-1β levels by inhibiting the TLR4/NF-κB pathway in microglia, thereby mitigating neuroinflammatory responses and promoting hippocampal BDNF expression, ultimately improving depressive-like behaviors in mice (Qian et al., 2024). These findings suggest that microbial metabolites derived from the gut microbiota may possess significant antidepressant potential. Therefore, integrating multi-omics analysis could provide novel insights into the molecular mechanisms underlying the antidepressant effects of *Limosilactobacillus fermentum*.

## 5 Conclusion

*Limosilactobacillus fermentum* WIS32 significantly alleviates LPS-induced depressive-like behaviors in mice by remodeling the gut microbiota structure, inhibiting neuroinflammation, and promoting neurotransmitter release via the microbiota-gut-brain axis (MGBA). This strain demonstrates excellent gastrointestinal tolerance, as well as antioxidant and antibacterial probiotic properties. It specifically upregulates the abundance of beneficial bacteria, such as *Bacteroides stercorirosoris* and *Massilimaliae timonensis*, while inhibiting the pathogenic uncultured *Streptococcus*. These microbial shifts subsequently lead to the upregulation of hippocampal 5-HT and BDNF levels and a reduction in the expression of peripheral and central TNF-*α*, IL-1β, and IL-6. This study provides a robust theoretical and experimental basis for the application of WIS32 as a potential microecological agent for the treatment of depression.

## Data Availability

The raw data have been deposited in the NCBI repository under accession number PRJNA1463616.
